# Unique Interplay between Sugar and Lipid in Determining the Antigenic Potency of Bacterial Antigens for NKT Cells

**DOI:** 10.1371/journal.pbio.1001189

**Published:** 2011-11-01

**Authors:** Enrico Girardi, Esther Dawen Yu, Yali Li, Norihito Tarumoto, Bo Pei, Jing Wang, Petr Illarionov, Yuki Kinjo, Mitchell Kronenberg, Dirk M. Zajonc

**Affiliations:** 1 Division of Cell Biology, La Jolla Institute for Allergy & Immunology, La Jolla, California, United States of America; 2 Department of Chemotherapy and Mycoses, National Institute of Infectious Diseases, Tokyo, Japan; 3 Department of Infectious Disease and Infection Control, Saitama Medical University, Saitama, Japan; 4 Division of Developmental Immunology, La Jolla Institute for Allergy & Immunology, La Jolla, California, United States of America; 5 School of Biosciences, University of Birmingham, Edgbaston, Birmingham, United Kingdom; National Jewish Medical and Research Center/Howard Hughes Medical Institute, United States of America

## Abstract

Invariant natural killer T (iNKT) cells are an evolutionary conserved T cell population characterized by features of both the innate and adaptive immune response. Studies have shown that iNKT cells are required for protective responses to Gram-positive pathogens such as *Streptococcus pneumoniae*, and that these cells recognize bacterial diacylglycerol antigens presented by CD1d, a non-classical antigen-presenting molecule. The combination of a lipid backbone containing an unusual fatty acid, vaccenic acid, as well as a glucose sugar that is weaker or not stimulatory when linked to other lipids, is required for iNKT cell stimulation by these antigens. Here we have carried out structural and biophysical studies that illuminate the reasons for the stringent requirement for this unique combination. The data indicate that vaccenic acid bound to the CD1d groove orients the protruding glucose sugar for TCR recognition, and it allows for an additional hydrogen bond of the glucose with CD1d when in complex with the TCR. Furthermore, TCR binding causes an induced fit in both the sugar and CD1d, and we have identified the CD1d amino acids important for iNKT TCR recognition and the stability of the ternary complex. The studies show also how hydrogen bonds formed by the glucose sugar can account for the distinct binding kinetics of the TCR for this CD1d-glycolipid complex. Therefore, our studies illuminate the mechanism of glycolipid recognition for antigens from important pathogens.

## Abstract 2

Structural and biophysical studies reveal the induced-fit mechanism underlying the stringent specificity of invariant natural killer T cells for unique glycolipid antigens from the pathogen *Streptococcus pneumoniae*.

## Author Summary

Invariant natural killer T (iNKT) cells are an evolutionarily conserved population of immune cells that recognize lipid antigens. A protein called a T cell receptor for antigen (TCR) on the surface of these iNKT cells recognizes lipids bound to a protein called CD1d on the surface of antigen-presenting cells. Here we describe the three-dimensional structure of the complex that forms between CD1d and the iNKT TCR together with a glycolipid antigen from the infectious bacterium *Streptococcus pneumoniae*, which is a common cause of bacterial meningitis in adults and is responsible for many other pneumococcal infections. We determined the three-dimensional structure of the complex by X-ray crystallography. The data obtained allow us to understand the structural requirements that make this glycolipid a potent antigen for iNKT cells, and why the TCR of these cells recognizes a particular combination of hexose sugar and diacylglycerol lipid. Moreover, by mutating CD1d and using biophysical methods to study the mutant protein complexes, we analyzed the role of the protein–protein interface between CD1d and the TCR and found that it plays an important role in the stability, but not the formation, of the trimolecular complex containing glycolipid antigen.

## Introduction

Invariant NKT cells (iNKT) are an evolutionarily conserved population of T lymphocytes able to respond to lipid antigens when presented by CD1d, a non-classical MHC class I–like molecule [1].

Antigen recognition by iNKT cells is mediated by a semi-invariant αβ T cell receptor (TCR) formed by a conserved Vα14-Jα18 rearrangement (Vα24-Vα18 in humans), and a limited panel of pairing β chains (Vβ8.2, Vβ7, Vβ2 in mouse; Vβ11 in humans). The number of antigens recognized by these cells has increased in the last few years, following the discovery of α-galactosylceramide (α-GalCer), the prototypical iNKT antigen [2]. Although the nature of the predominant self-antigens recognized by iNKT cells still remains controversial, important progress has been made in describing the microbial antigens recognized by this cell type. Glycosphingolipids from *Sphingomonas* spp. and diacylglycerol (DAG) ligands from *Borrelia burgdorferi*, the causative agent of Lyme disease, were identified to stimulate iNKT cells in a CD1d/TCR-dependent manner [3]–[7]. As *Sphingomonas* spp. and *B. burgdorferi* are not responsible for widespread or lethal diseases, we considered it possible that more pathogenic organisms also express iNKT antigens, which would account for the highly conserved nature of the CD1d-iNKT TCR interaction. Indeed, recent studies identified the structures of DAG compounds from the highly pathogenic *Streptococcus pneumoniae* (*S. pneumoniae*) and *Group B streptococcus* (GBS), which were able to stimulate iNKT cells [8]. In vitro and in vivo assays demonstrated surprisingly strict requirements for these antigens in activating iNKT cells. The most potent *S. pneumoniae* antigen, Glc-DAG-s2, is characterized by having a *sn*-3 linked glucose, a *sn*-1 linked palmitic acid (C16∶0), and most importantly, the presence of *cis*-vaccenic acid (C18∶1, n-7) in position *sn*-2 of the glycerol moiety. This uncommon fatty acid was required for significant activity, since the positional isomer with a vaccenic acid in position *sn*-1 failed to elicit a strong activation, as did the homologous compounds containing an oleic acid (C18∶1, n-9). Moreover, the same antigen showed the ability to stimulate both mouse and human iNKT cells, unlike the previously characterized DAG antigens from *B. burgdorferi* [6]. Interestingly, previous studies showed that glucose-containing glycolipids are relatively weaker antigens compared to the one containing galactose or galacturonic acid [2],[3],[9],[10], while the glucose isomer of the *B. burgdorferi* glycolipid 2c (BbGL-2c) is not antigenic at all [6]. It is therefore surprising that the glucose-containing *S. pneumoniae* Glc-DAG-s2 is such a potent antigen in eliciting iNKT cell responses.

In order to determine the molecular basis for the stringent structural requirements for recognition of the *S. pneumoniae* antigen Glc-DAG-s2, and to further analyze the mechanism of the mouse CD1d (mCD1d)-iNKT TCR complex formation, we determined the structure of the mCD1d-Glc-DAG-s2-iNKT TCR complex by X-ray crystallography and we analyzed the role of the F′ roof in the formation and stability of mCD1d-iNKT TCR complexes. Our data show how the combination of *cis*-vaccenic acid and glucose is required for the formation of novel protein-antigen contacts, resulting in the relatively strong affinity of this ligand for the iNKT TCR.

## Results

### mCD1d-Glc-DAG-s2-TCR Structure

**Figure 1:**
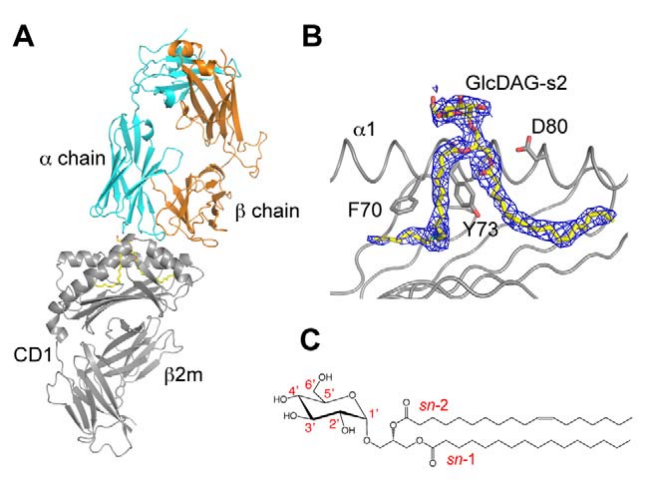
Structure of the mCD1d-Glc-DAG-s2-TCR complex. (A) Cartoon representation of the mCD1d-Glc-DAG-s2-TCR trimolecular complex with the CD1d/β2m protein in grey, and the TCR α and β chains in cyan and orange, respectively. The Glc-DAG-s2 antigen is shown as yellow sticks, tucked at the interface between CD1d and the TCR. (B) Side-view of the antigen-binding groove in the mCD1d-Glc-DAG-s2-TCR complex with the DAG antigen shown in sticks and the α2 helix removed for clarity. The 2Fo−Fc electron density map is contoured at 1σ and shown as a blue mesh around the glycolipid ligand. Several mCD1d residues interacting with the lipid are depicted. (C) Chemical structure of the Glc-DAG-s2 antigen with vaccenic acid in the *sn*-2 position.

Previous biophysical analysis of the kinetics of interaction of the mCD1d-Glc-DAG-s2 complex with the iNKT TCR revealed a TCR interaction with a low micromolar affinity [8]. Overall, Glc-DAG-s2 complexes with mouse CD1d are characterized by a comparable but slightly higher affinity for the iNKT TCR compared to those containing the other known bacterial DAG antigen BbGL-2c (*K*_D_ of 4.4 and 6.2 µM, respectively), consistent with the similar antigenic potencies of these compounds [8],[11]. However, complexes containing Glc-DAG-s2 have significantly different binding kinetics compared to those containing BbGL-2c, with Glc-DAG-s2 showing considerably slower association and dissociation rates. In order to investigate the molecular basis for this different kinetic behavior and the role of the unique structural features of this ligand in determining its antigenicity, we determined the crystal structure of the mCD1d-Glc-DAG-s2-iNKT TCR complex at 2.7 Å resolution (Figure 1, Table 1).

**Table 1:** Data collection and refinement.

Statistics	mCD1d-Glc-DAG-s2-TCR
**Data collection**	
Space group	*C*222_1_
Cell dimension	
*a, b, c,* (Å)	78.1, 190.7, 150.9
α, β, γ (°)	90.0, 90.0, 90.0
Resolution range (Å) [outer shell]	44.5–2.70 [2.85–2.70]
No. reflections	31,376
R_merge_ (%)	13.5 [53.0]
R_pim_ (%)	7.5 [30.0]
R_meas_ (%)	15.5 [61.2]
Multiplicity	4.0 [4.0]
Average I/σI	7.1 [2.4]
Completeness (%)	99.8 [99.9]
**Refinement statistics**	
No. atoms	6,554
Protein	6,276
Ligand	53
Carbohydrate	80
Waters	145
R/R_free_	0.203/0.257
Ramachandran plot (%)	
Favored	97.1
Allowed	100.0
R.m.s. deviations	
Bonds (Å)	0.010
Angles (°)	1.275
B-factors (Å^2^)	
Protein	37.4
Ligand	44.6
Carbohydrate	57.3
Waters	30.4

**Figure 2:**
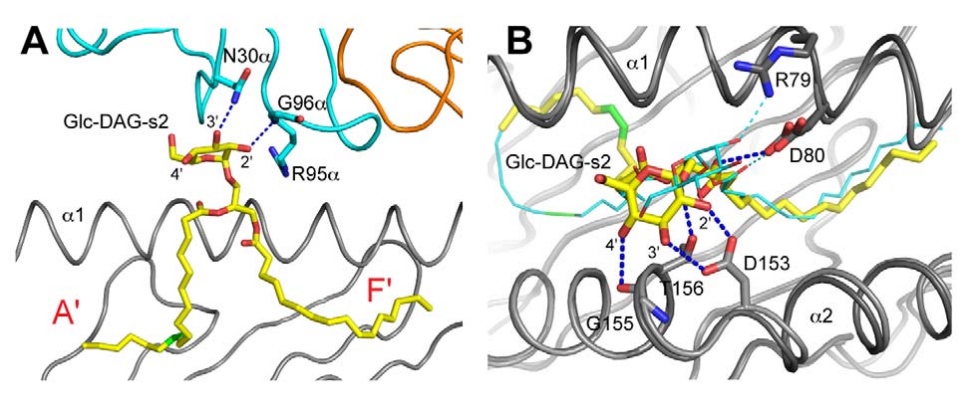
Binding of Glc-DAG-s2 to mCD1d and the TCR. (A) Contacts between Glc-DAG-s2 and the iNKT TCR. The conserved hydrogen bonds, involving key residues on CDR1α and CDR3α of the TCR, are shown as dashed blue lines. Glc-DAG-s2, yellow; mCD1d heavy chain, grey; TCR α chain, cyan; TCR β chain, orange. Top (B) view of the mCD1d interactions with Glc-DAG-s2 in the presence (grey, Glc-DAG-s2 in yellow) or absence (dark grey, Glc-DAG-s2 in cyan) of the TCR. Hydrogen bond interactions between mCD1d residues and the polar moieties of Glc-DAG-s2 are indicated with blue dashed lines for the ternary complex and cyan for the mCD1d-Glc-DAG-s2 complex.

The structure shows the conserved “parallel" docking mode of the iNKT TCR on the CD1d-ligand complex (Figure1A) [12]–[14]. As a consequence of this unique binding mode, the TCR α chain mediates the majority of the contacts with the CD1d-Glc-DAG-s2 complex, with additional contacts with CD1d provided by the CDR2β, CDR3β, and, to a lesser extent, the CDR1β loops (Table S1). Well-defined, unbiased density was present for the ligand, superior to what has been observed for the mCD1d-Glc-DAG-s2 complex in absence of the TCR [8], suggesting that the ligand adopts a more rigid and ordered conformation upon TCR binding (Figure 1B, Figure S1). Similar to the antigens previously characterized, the TCR CDR1α and CDR3α loops exclusively mediate contacts between the TCR and the antigen (Figure 2A). In particular, the TCR recognizes the 2′-OH and 3′-OH positions of the hexose ring via H bonds with Gly96 and Asn30 on the α chain, highlighting the importance of these two hydroxyl groups on the antigen in the formation of the complex. However, due to the presence of a glucose on Glc-DAG-s2, the 4′ hydroxyl group is no longer able to interact with Asn30 on the α chain, in contrast to other galactose-containing glycolipids. Previous studies showed that the contacts between the ligand and the iNKT dominate the initial association phase of the interaction [9]. The loss of an H bond at the ligand-TCR interface, although not sufficient to abolish the binding of the iNKT TCR to the mCD1d-Glc-DAG-s2 complex, is therefore likely to decrease the association rate.

### Induced Fit of the Glycolipid upon TCR Binding

When the structures of the mCD1d-Glc-DAG-s2 complex in the presence or absence of the TCR are compared, important conformational changes are observed for the ligand (Figure 2B). Consistent with what was observed for the mCD1d-Glc-DAG-s2 structure in the absence of the TCR [8], the *sn*-2 vaccenic acid is bound in the A′ pocket while the *sn*-1 palmitic acid occupies the F′ pocket. However, while in the absence of the TCR the vaccenic acid encircles the A′ pole in a clockwise manner, the opposite orientation is preferred in the ternary complex, although residual density also suggests some equilibrium between the two orientations. Moreover, upon TCR ligation, the glucose moiety is shifted by about 30 degrees clockwise around its glycosidic bond to assume a position at the center of the binding groove as observed for other TCR-bound glycolipid antigens (Figure 2B). Similar to what has been described for BbGL-2c, this conformational change requires the breaking of several contacts with CD1d and the formation of new hydrogen bonds with the α2 helix of CD1d and the TCR α chain. In particular, a hydrogen bond with Arg79 on the α1 helix is lost while new polar contacts are formed with Asp153 and Thr156 on the α2 helix upon TCR binding, resulting in a final orientation conserved among α-linked sugars [13]–[15]. As proposed for BbGL-2c, these conformational changes likely contribute to the slower association rate of the TCR when binding DAG microbial antigen-mCD1d complexes compared to sphingolipid-containing antigens [14]. However, the presence of glucose on Glc-DAG-s2 results in an additional H bond between the antigen and the backbone of the α2 helix of CD1d, involving the carbonyl group of glycine 155 (3.2 Å, Figure 2B). Because this contact stabilizes a favorable binding conformation of the antigen in the binding groove, it is likely that this feature is contributing to the slower complex dissociation observed for this ligand compared to BbGL-2c, as the TCR has to invest less energy to lock the glucose into place. Moreover, comparison of the mCD1d-TCR molecular contacts in the two DAG antigens ternary complexes revealed a slightly optimized interface for Glc-DAG-s2 compared to BbGL-2c (involving in particular additional salt bridges between CDR3α and CDR2β residues with mCD1d; Table S1; [14]), which could have a further stabilizing effect on the dissociation rate of the ternary complex.

### Interplay between the Sugar and Lipid for Determining Antigenicity

**Figure 3:**
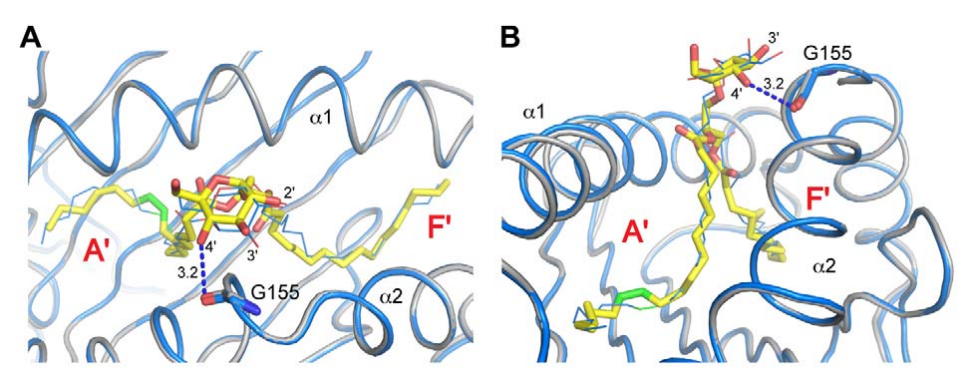
Binding of Glc-DAG-s2 and BbGL-2c to mCD1d. Top (A) and side (B) view of the mCD1d interactions in the presence of the TCR. Glc-DAG-s2 is shown in yellow and BbGL-2c in blue and thin lines with the respective CD1d structure in grey and blue. The novel hydrogen bond formed between G155 on the α2 helix and the 4′-OH of Glc-DAG-s2 is indicated with a blue dashed line with its distance expressed in Å. The unsaturations in the alkyl chains are shown in green and sit in a similar region of the A′ pocket.

When the structures of the ternary complexes of the DAG antigens Glc-DAG-s2 and BbGL-2c are compared (Figure 3), it is interesting to note how the unsaturations present on the respective vaccenic and oleic acids are localized in the same portion of the mCD1d A′ pocket, suggesting a preference for this region of the groove for binding unsaturated alkyl chains. Consistent with this, it was previously noted that the presence of unsaturations improved the stability of the mCD1d-glycolipid complex, possibly due to the kink introduced in the alkyl chain by the unsaturated bonds, which could nicely sit at the bottom of the channel connecting the A′ pocket with the protein surface [16],[17]. Assuming that an unsaturated fatty acid would preferentially bind in the A′ pocket, in the positional isomer of Glc-DAG-s2 having the vaccenic acid at the *sn*-1 position, this would result in a reversed orientation of the glycerol backbone in the binding groove. The reversed glycerol orientation would cause an unfavorable positioning of the glucose head, therefore explaining the lack of antigenic activity for this compound [8].

**Figure 4:**
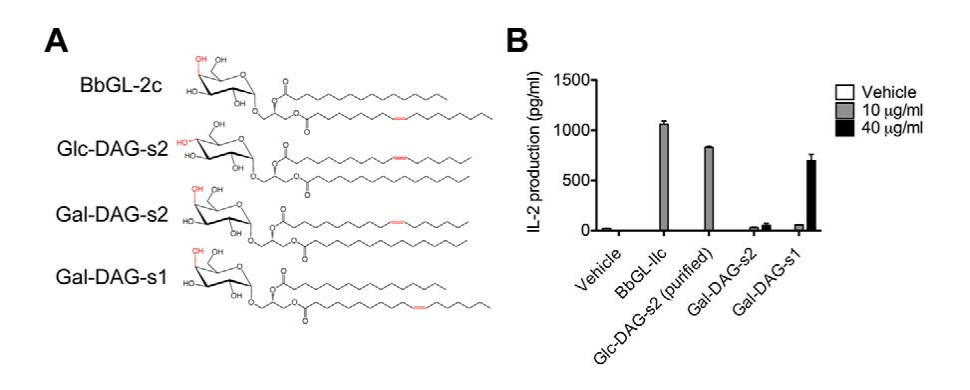
Stringent specificity requirements for recognition of the *S. pneumoniae* iNKT antigens. (A) Chemical structure of bacterial antigens BbGL-2c and Glc-DAG-s2 together with galactose-containing versions of Glc-DAG-s2. The 4′ hydroxyl group, which differs in orientation between glucose and galactose and the unsaturations of the antigens, is shown in red. (B) Antigen presenting cells expressing mCD1d were pulsed with the indicated amounts of each compound and were then cultured with iNKT cell hybridoma 1.2. IL-2 amounts in the culture supernatant are shown. The error bars indicate the SEM of triplicate measurements and the data are representative of four separate experiments. Due to their relatively strong response, BbGL-2c and Glc-DAG-s2 were tested exclusively at 10 µg/ml.

A superposition of the mCD1d-Glc-DAG-s2 and mCD1d-BbGL-2c complexes in presence of the TCR shows how the two hexose groups are oriented slightly differently at the opening of the binding groove (Figure 3A). The lack of activity of the Glc-DAG-s2 analog containing an oleic acid in place of vaccenic acid suggests that the vaccenic acid is required for a more favorable orientation of the exposed glucose, possibly enhancing the ability of the glucose moiety to contact Gly155 and therefore positioning it in a more stable fashion in the correct orientation for TCR recognition. Galactose and glucose differ only with regard to the orientation of the 4′ hydroxyl on the hexose sugar ring, with the axial orientation for galactose and the equatorial orientation, i.e., closer to the plane of the ring, for glucose. Despite the structural similarity of the two sugars, intriguingly, the galactose-containing version of the *S. pneumoniae* DAG glycolipid antigen with a *sn*-2 vaccenic acid, called Gal-DAG-s2, was not able to activate mouse iNKT cell hybridomas (Figure 4). A drastically reduced response was also observed for the Gal-DAG-s1 ligand. This indicates that the presence of vaccenic acid in the A′ pocket of the mCD1d binding groove does not automatically confer antigenicity. When the stereochemistry of the 4′ carbon is inverted, converting the glucose of Glc-DAG-s2 into galactose, it becomes clear that the 4′-OH group will be too distant (4.3 Å, Figure S2) to engage Asn30 on the CDR3α loop, while at the same time losing the contact with Gly155 on the α2 helix of mCD1d. Even if a further reorientation of the galactose sugar by the iNKT TCR were possible, this would require an additional energetic toll, suggesting a rationale for the reduced activity of the Gal-DAG-s2 compound. It is therefore evident that the combination of the uncommon vaccenic acid and the glucose sugar, which is relatively weak in the context of other DAG antigens and glycosphingolipids, is required for the optimal positioning of the Glc-DAG-s2 antigen in the mCD1d binding groove for TCR recognition.

### The mCD1d F′ Roof Affects Recognition by the iNKT TCR

**Figure 5:**
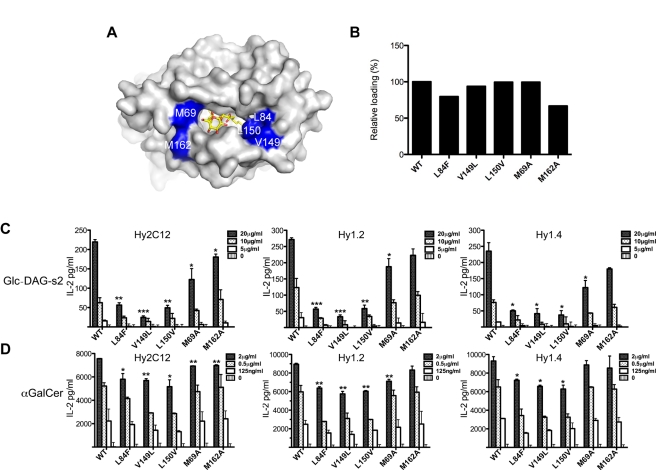
Antigen presentation by F′ roof mutants. (A) Surface of the mCD1d-Glc-DAG-s2 structure in the presence (grey, Glc-DAG-s2 in yellow) of the iNKT TCR. The residues mutated for further analysis are shown in blue. (B) Relative antigen loading efficiencies by the mCD1d mutants as determined by surface plasmon resonance. Values are expressed as percentages with the WT protein set at 100%. The ability of CD1d mutants to present Glc-DAG-s2 (C) and α-GalCer (D) was analyzed in a cell free antigen presentation assay using CD1d coated plates and the Vα14*i* NKT cell hybridomas Hy2C12, Hy1.2, Hy1.4. As a measure of direct iNKT cell activation IL-2 was measured in the culture supernatant by ELISA. Each bar shows mean + SD from duplicate wells and is representative of two independent experiments.

The structures of the iNKT cell TCR in complex with different mCD1d-microbial antigens complexes showed how the iNKT TCR is able to induce conformational changes in both the ligand and mCD1d upon complex formation [14]. In particular, the insertion of amino acid Leu99α, located on the CDR3α loop of the iNKT TCR, between residues Leu84, Val149, and Leu150 above the F′ pocket of mCD1d, resulted in several new van der Waals (VdW) contacts and the formation of a hydrophobic surface above the pocket (F′ roof). Consistent with this, a comparison of mCD1d-Glc-DAG-s2 structures before and after TCR binding reveals that the F′ roof also is formed for this antigen upon TCR binding (Figure 5A).

In order to understand and validate the role of the F′ roof in the formation of a more stable CD1d-Glc-DAG-s2-TCR complex, we mutated selected residues involved in the formation of the roof and characterized the ability of the mutated mCD1d proteins to stimulate iNKT cell hybridomas. As a complete removal of the F′ roof would likely result in an abrogation of binding, as demonstrated by the loss of function mutation of L99α in the TCR to alanine [18], we chose mutations of the relevant position in mCD1d that would maintain the hydrophobic nature of their side chains, in order to perturb the F′ roof area without making a too drastic change. We therefore used site-directed mutagenesis to generate the following mCD1d substitutions: Leu84Val, Leu84Phe, the latter mimicking the human homolog, Val149Leu and Leu150Val, together with two control mutants, Met69Ala and Met162Ala from the area above the A′ pocket (Figure 5A). Interestingly, we obtained drastically reduced expression yields for the Leu84Val mutant, and this construct was not tested further. The iNKT cell hybridomas Hy2C12 (bearing the Vα14Vβ8.2 TCR used in our structural studies), Hy1.2 (also Vα14Vβ8.2), and Hy1.4 (expressing a less common Vα14Vβ10 TCR) were tested for their ability to respond to mCD1d-glycolipid complexes in an antigen presenting cell-free assay using mCD1d-coated plates. IL-2 secretion provided a measure of TCR stimulation. We stimulated the cells with either Glc-DAG-s2 or α-GalCer, the prototypical iNKT cell antigen that induces a preformed F′ roof on mCD1d [19]. When loaded with Glc-DAG-s2 or α-GalCer, all the mutants showed a reduced ability to stimulate the hybridoma (Figure 5). In particular, the mutants Leu84Phe and Val149Leu abrogated iNKT cell activation, while a slightly reduced activity was observed with the Leu150Val mutant.

As the reduced response could be the consequence of impaired loading of these antigens on mCD1d, we measured the loading efficiency of α-GalCer on each mutant by surface plasmon resonance (SPR) using a monoclonal antibody (L363 [20]) specifically reactive to complexes of mCD1d with α-GalCer and analogs (Figure 5B). Although the Leu84Phe and Met62Ala mutants showed lower levels of antigen loading compared to wild type mCD1d, loading on the mutated mCD1d proteins was never below 65% of the wild type control, and does not appear to correlate directly with the ability of the mutated proteins to stimulate the iNKT cell hybridoma. Although the lack of an antibody able to recognize the mCD1d-Glc-DAG-s2 complex did not allow us to assess the loading of this antigen onto the mCD1d mutants, we believe it is unlikely that the two ligands have radically different loading efficiencies, suggesting a critical role of the area above the F′ pocket in the TCR interaction with mCD1d/Glc-DAG-s2 and mCD1d/α-GalCer.

### Mutation of F′ Roof Residues Affects Preferentially the Stability of the mCD1d-iNKT TCR Complex

**Figure 6:**
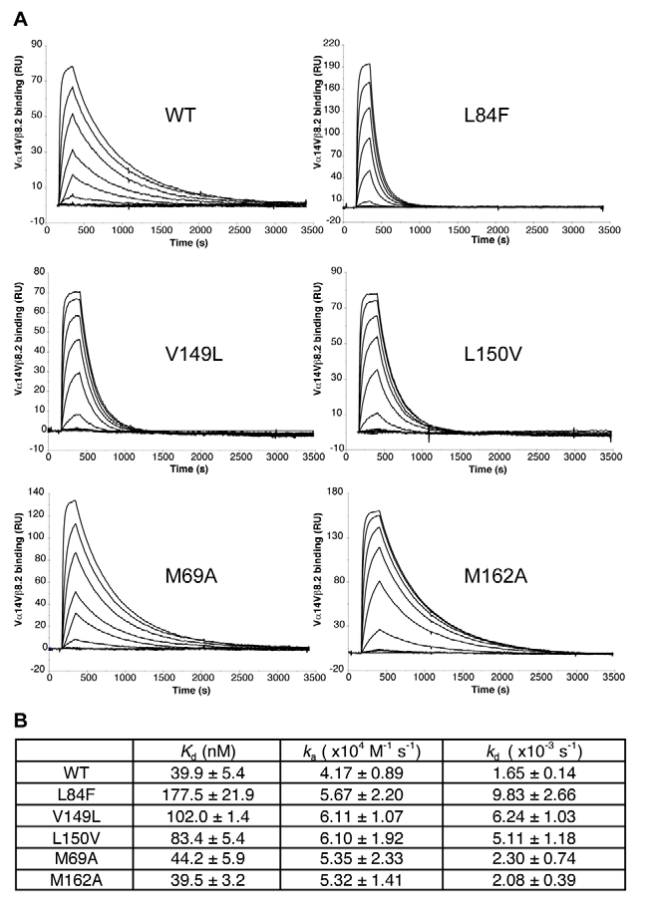
F′ roof mutations affect the stability of the mCD1d-iNKT cell TCR complex. (A) Binding response of a Vα14Vβ8.2 TCR to immobilized mouse CD1d loaded with α-GalCer as measured by surface plasmon resonance. Binding of increasing concentrations of the TCR is shown for each mutant as black lines. (B) Kinetic parameters measured for WT and mutants proteins.

We previously hypothesized that the formation of the F′ roof on CD1d affects the stability of the CD1d interaction with the iNKT TCR [14]. To validate this hypothesis we measured the effect of the F′ roof mutants on the binding kinetics of the mCD1d-iNKT TCR complex (Figure 6). Because of the relatively weak antigenicity of Glc-DAG-s2 compared to α-GalCer, the latter was chosen for SPR analysis. A comparison of the affinities shows how all the F′ roof mutants have weaker affinities for the iNKT TCR, Leu84Phe being the weakest with a ∼4-fold reduction compared to the wild type protein (Figure 6) while the two control mutants showed affinities and kinetics similar to the wild type protein. Strikingly, the differences in affinities between mCD1d F′ roof variants derive mainly from faster dissociation rates for the mutant complexes, while the association rates are minimally affected by perturbation of the F′ roof. Mutation of residues Val149 and L150 appear less disruptive than mutation of Leu84, as the Leu84Phe has the fastest dissociation rate. Taken together, these data suggest that the F′ roof is critical in determining the dissociation rate and therefore the stability of the mCD1d-TCR complex.

## Discussion

Activation of iNKT cells can result as a consequence of TCR-independent, IL12-dependent signals and/or through the recognition of self and foreign antigens by its semi-invariant TCR, with the latter mechanism playing an important role in modulating the overall response [21]. The recent discovery that highly pathogenic Gram-positive bacteria express antigens recognized by both mouse and human iNKT cells [8] therefore opens important perspectives for the development of therapeutic agents against pneumonia and meningitis, while also suggesting a potential rationale for the conserved features of the CD1d-TCR interaction among different mammalian species.

Surprisingly, the *S. pneumoniae* Glc-DAG-s2 antigen presents unusual chemical features in both its lipidic and polar portions when compared to the previously characterized iNKT cell microbial antigens from *Sphingomonas* spp. or *Borrelia burgdorferi*. Instead of the α-galactose or α-galacturonic acid found on these antigens, and on the prototypical antigen α-GalCer, the otherwise weaker α-glucose is found in *S. pneumoniae* as well as in another gram-positive pathogen, GBS. Furthermore, the sugar is α-linked to a DAG backbone containing on position *sn*-2 the uncommon *cis*-vaccenic acid. Despite containing a glucose sugar, Glc-DAG-s2 was at least as active as the *Borrelia* BbGL-2c lipid in activating a mouse iNKT cell hybridoma and it showed similar antigenic potency in vivo (Figure 4B; [8]). Interestingly, we show here and in the previous studies that these unusual features are required together for the glycosylated DAG lipid to have any measurable antigenic potency. These stringent requirements were correlated with unusual binding kinetics compared to the *B. burgdorferi* DAG antigens, characterized by slow association and slow dissociation rates of the mCD1d-ligand complex to the iNKT TCR [8]. The structure of the mCD1d-Glc-DAG-s2-TCR complex presented here, together with the other studies we have carried out, allow us to understand the stringent chemical requirements, as well as the distinct TCR binding kinetics, in the recognition of the DAG antigens from these highly pathogenic bacteria.

As for the other DAG antigen, BbGL-2c, the iNKT TCR is able to induce conformational changes on both the Glc-DAG-s2 ligand and mCD1d (Figures 2 and 5A), which result in a conserved binding mode, as well as a weaker affinity, typical of the DAG antigens compared to their glycosphingolipid counterparts [14],[22]. However, due to the presence of glucose, with a different conformation of its 4′ hydroxyl group compared to galactose, a hydrogen bond with the CDR1α of the TCR is lost, while a new contact with Gly155 on the α2 helix of CD1d is formed. While this alteration does not translate to an overall change in TCR affinity at equilibrium, it has profound effects on the kinetics of binding of the antigen complex with mCD1d by the iNKT cell TCR. Previous studies showed that contacts between the ligand and the TCR dominate the initial association phase, while protein-protein (and ligand-protein) contacts affect the stability of the complex [9]. Therefore, the loss of a contact with the α chain of the TCR can account for the slower TCR association rate exhibited by this ligand. Interestingly, the iNKT TCR appears to be especially sensitive to the conformation of the 4′-OH group, with glucose-containing antigens showing generally reduced potency (in terms of cytokine release by iNKT cells) [9],[10] and preferential proliferation of Vβ7+ cells [10] compared to α-GalCer. Furthermore, the consequent locking of the glucose head in the favorable position following TCR engagement, described here for Glc-DAG-s2, likely contributes to a slower dissociation. These novel contacts rely on the presence of both the vaccenic acid and glucose, as the variants with an oleic acid in place of the vaccenic acid, or a galactose replacing the glucose, are considerably less active. Consistent with this, a model of Gal-DAG-s2 suggests that the presence of an axial 4′ hydroxyl would be located in an unfavorable position for recognition by the iNKT TCR (Figure S2). Moreover, the antigenicity of the ligand requires vaccenic acid to be in the *sn*-2 position of the ligand in order to orient correctly the glucose for recognition by the TCR. Interestingly, these structural requirements do not appear to be influenced by the variable CDR3β loop, as three different hybridomas responded to Glc-DAG-s2 at comparable levels (Figure 5C).

Glc-DAG-s2 also stimulates human iNKT cells [8] but is not clear whether the same stringent requirements observed in mouse are conserved in the human CD1d-TCR interaction as no structural information is available on the modality of recognition of DAG antigens by the human iNKT TCR. Clearly, more work has to be done to illuminate the structural basis of microbial DAG recognition by human iNKT cells.

Consistent with a model in which the TCR contacts first the ligand and subsequently CD1d, our mutational data also show that the protein-protein interface above the F′ pocket is critical for the interaction, and specifically, that this region determines the dissociation rate, and therefore the stability, of the mCD1d-TCR complex (Figure 6). Interestingly, the mechanism of antigen recognition by the iNKT TCR appears to be radically different to what has been observed for MHC-TCR interactions, where the TCR first contacts residues on the antigen presenting molecule and only a later stage contacts the antigen itself [23].

The extensive amount of structural and biochemical information recently collected on the interaction between CD1d and the iNKT TCR is consistent with the idea of the iNKT TCR as a pattern recognition receptor [10],[12]–[14],[18]. While the *S. pneumoniae* antigen follows the typical pattern of an α-linked sugar to a diacyl backbone, the data presented here show clearly that, within this pattern, stringent requirements are still in place. In particular, the Glc-DAG-s2 ligand exemplifies the case of a relatively weak hexose and an uncommon alkyl chain synergistically contributing to the potency of an iNKT antigen.

## Materials and Methods

### Protein Expression and Purification

The expression and purification methods of fully glycosylated mouse CD1d/β2m heterodimer proteins were reported previously [11]. Mouse TCR refolding was performed according to previously reported protocols [14] with minor modifications. 64 mg of α chain and 96 mg of β chain inclusion bodies were mixed together and added drop wise to 1 L refolding buffer (50 mM Tris-HCl, 0.4 M L-arginine, 5 M urea, 2 mM EDTA, 5 mM reduced glutathione, 0.5 mM oxidized glutathione, 0.2 mM PMSF, pH 8.0 at RT) two times. The refolding mix was dialyzed twice against 18 L dialysis buffer 1 (10 mM Tris-HCL, 0.1 M urea, pH 8.0) for 16 h and then once against 18 L of 10 mM Tris-HCl pH 8.0 for 24 h. The refolded TCR proteins were purified by MonoQ 5/50 GL (GE Healthcare) using a linear NaCl gradient (0–300 mM NaCl) followed by size exclusion chromatography using a Superdex S200 10/300 GL (GE Healthcare) in 50 mM Hepes pH 7.5, 150 mM NaCl.

### Glycolipid Loading and Ternary Complex Formation

The synthetic DAG ligand Glc-DAG-s2 was synthesized as previously reported [8] and dissolved at 4 mg/ml in DMSO. mCD1d was incubated overnight with 3–6 molar excess of Glc-DAG-s2 in presence of 0.05% Tween-20 and 100 mM Tris-Cl pH 7.0. Glc-DAG-s2 loaded CD1d was purified by size exclusion chromatography first and then incubated with equimolar amount of TCR for 30 min without further purification. The complex was concentrated to 4.8 mg/ml for crystallization.

### Crystallization and Structure Determination

Crystals of mCD1d-Glc-DAG-s2-TCR complexes were grown at 22.3°C by sitting drop vapor diffusion while mixing 0.5 µl protein with 0.5 µl precipitate (17% polyethylene glycol 3350, 8% v/v Tacsimate pH 5.0). Crystals were flash-cooled at 100 K in mother liquor containing 20% glycerol. Diffraction data were collected at the Stanford Synchrotron Radiation Laboratory (SSRL) beamline 7.1 and processed with the iMosflm software [24]. The mCD1d-Glc-DAG-s2-TCR crystal belongs to space group *C*222_1_ with cell parameters *a* = 78.1 Å; *b* = 190.7 Å; *c* = 150.9 Å. The asymmetric unit contains one mCD1d-glycolipid-TCR molecule with an estimated solvent content of 55.0%. The structures were determined by molecular replacement using MOLREP as part of the CCP4 suite [25],[26] using the protein coordinates from the mCD1d-iGb3 structure (PDB code 2Q7Y) [27], followed by the Vα14Vβ8.2 TCR [14] (from PDB code 3O8X) as the search model. When a MR solution containing both mCD1d and TCR was obtained, the model was rebuilt into σ_A_-weighted 2*F_o_*–*F_c_* and *F_o_*–*F_c_* difference electron density maps using the program COOT [28]. Maximum-likelihood restrained refinement coupled with TLS refinement was performed in REFMAC [29] with five anisotropic domains (α1-α2 domain of CD1d, including carbohydrates and glycolipid, α3-domain, β2m, variable domains and constant domains of the TCR). The quality of the model was evaluated with the program Molprobity [30] and the validation tools available in COOT. Shake-omit maps were generated by removing the ligand from the structure and randomly perturbating the coordinates, occupancy, and B-factor of each atom by 0.2 Å, 0.05 units, and 20 Å^2^, respectively, with the software Moleman2 [31]. The resulting structure was then refined with the software REFMAC as described earlier. Data collection and refinement statistics are presented in Table 1. Coordinates and structure factors have been deposited in the Protein Data Bank under accession code 3TA3.

### Generation of mCD1d Mutants

Mouse CD1d mutants were generated using Quick Change II Site-Directed Mutagenesis Kit (Stratagene, Agilent Technologies) according to the manufacturer's instructions with the primers indicated below. Mutated constructs were purified with the Qiagen Miniprep Kit (Qiagen) and the presence of the mutation confirmed by sequencing. The mutated birA-tag mCD1d/β2m were expressed and purified using the same method described above for mCD1d/β2m.

Primer sequences: L84V 5′-ttaccagggacatacaggaagtagtcaaaatgatgtcacc-3′; L84V_antisense 3′-aatggtccctgtatgtccttcatcagttttactacagtgg-5′; L84F 5′-accagggacatacaggaattcgtcaaaatgatgtcacc-3′; L84F_antisense 3′-tggtccctgtatgtccttaagcagttttactacagtgg-5′; V149L 5′-cttggttagacttgcccatcaaattgctcaacgctg-3′; V149L_antisense 3′-gaaccaatctgaacgggtagtttaacgagttgcgac-5′; L150V 5′-cttgcccatcaaagtggtcaacgctgatcaagg-3′; L150V_antisense 3′-gaacgggtagtttcaccagttgcgactagttcc-5′; M69A 5′-gtgggagaagttgcagcatgcgtttcaagtctatcgagtc-3′; M69A_antisense 3′-gtgggagaagttgcagcatgcgtttcaagtctatcgagtc-5′; M162A 5′-caagtgcaaccgtgcaggcgctcctgaatgacacct-3′; M162A_antisense 3′-caagtgcaaccgtgcaggcgctcctgaatgacacct-5′.

### Cell Lines and Culture Conditions

A20/CD1d cells are derived from murine B cell lymphoma A20 (American Type Culture Collection, Rockville, MD), with stable expression of wild type mouse CD1d [7],[32]. A20/CD1d and *i*NKT hybridomas cell lines Hy2C12, Hy1.2, and Hy1.4 were cultured in RPMI 1640 medium supplemented with 2 mM L-glutamine, 100 mg/ml each of penicillin and streptomycin, 50 mM 2-mercaptoethanol, and 10% FBS.

### A20 Lipid Antigen Presentation Assay

Mouse iNKT cell hybridoma 1.2 (Vα14/Vβ8.2) has been described previously [3],[6]. 1×10^6^ A20/CD1d cells expressing wild-type mCD1d were cultured in complete medium containing indicated amounts of lipid antigens or vehicle (56 mg/ml sucrose, 7.5 mg/ml histidine, and 5 mg/ml Tween-20 [pH 7.2]) overnight. On the second day of culture, A20/CD1d were collected, washed thoroughly, and 1×10^5^ APCs were seeded in the presence of 5×10^4^ iNKT cell hybridomas per well in a 96-well plate for 24 h, and IL-2 in the supernatant was measured by ELISA according to the manufacturer's instructions (BD Biosciences).

### APC Free Antigen Presentation Assay

Stimulation of mouse iNKT cell hybridomas on microwell plates coated with soluble mCD1d was carried out according to published protocols [3],[6],[33], with a few modifications. Briefly, the indicated amounts of compounds were incubated for 24 h in microwells that had been coated with 1.0 µg of mCD1d. After washing, 5×10^4^ iNKT cell hybridoma cells were cultured on the plate for 16 h, and IL-2 in the supernatant was measured by ELISA according to the manufacturer's instructions (R&D systems).

### Surface Plasmon Resonance

Surface Plasmon Resonance studies using a refolded and biotinylated Vα14Vβ8.2 TCR were carried out as previously reported [11] with 300–500 response units (RU) of biotinylated mCD1d-vehicle or mCD1d-ligand immobilized on the chip. Serial dilutions of Vα14Vβ8.2 were injected with increasing concentrations (0.002–1.25 µM) over a streptavidin CAPture chip (GE Healthcare). The experiment was performed twice. Loading efficiency was measured by immobilizing the biotinylated mCD1d-ligand (after incubation for 16 h in the presence of 1 µg/ml of α-GalCer) complex on a CAPture chip (400–500 RU) followed by the injection of a saturating concentration (1 µM) of the Fab portion of the mCD1d-α-GalCer specific antibody L363 [20]. 100% glycolipid loading efficiency is achieved when the increase in RU upon Fab binding is equal to the RU of CD1d-glycolipid coated on the chip, as mCD1d-glycolipid and Fab have a comparable molecular weight.

## Supporting Information

Figure S1 Shake omit map of the Glc-DAG-s2 ligand. Side (A) and top (B) view of the mCD1d binding groove with the ligand in yellow. A shake-omit Fo-Fc map contoured at 2σ is shown as a green mesh around the ligand.
(TIF)

Figure S2 Modeling of Gal-DAG-s2 in the ternary complex. Detailed view of the Gal-DAG-s2 ligand at the mCD1d-TCR interface with the ligand in green, mCD1d in grey, and the iNKT TCR α chain in cyan. The different position of the 4′-OH group for Glc-DAG-s2 is shown in yellow for comparison. Distances between the 4′-OH group of the antigen and Asn30α on the TCR and Gly155 on mCD1d are shown as dashed lines with the corresponding length expressed in Å.
(TIF)

Table S1 Molecular contacts in the TCR complex. The program CONTACT [26] was used to analyze the molecular interactions within the complexes. Cutoffs of 4 Å (van der Waals interactions), 3.5 Å (hydrogen bonds), and 4.5 Å (salt bridges) were applied.
(DOC)

## Abbreviations

α-GalCer: α-galactosylceramide
BbGL-2c: *Borrelia burgorferi* glycolipid 2c
β2m: beta-2-microglobulin
CDR: complementarity determining regions
DAG: diacylglycerol
GalAGSL: galacturonosyl-glycosphingolipid from *Sphingomonas* spp
Glc-DAG-s2: glucose diacylglycerol lipid from *Streptococcus pneumoniae* with a vaccenic acid in position *sn*-2
iNKT: invariant Natural Killer T
k_a_: association rate constant
k_d_: dissociation rate constant
K_D_: equilibrium dissociation constant
mCD1d: mouse CD1d
