# Supplementary figures and images for: Unique Interplay between Sugar and Lipid in Determining the Antigenic Potency of Bacterial Antigens for NKT Cells
Source: PLoS Biol. 2011 Nov 1;9(11):e1001189. doi: 10.1371/journal.pbio.1001189 (PMC3206013; doi:10.1371/journal.pbio.1001189)

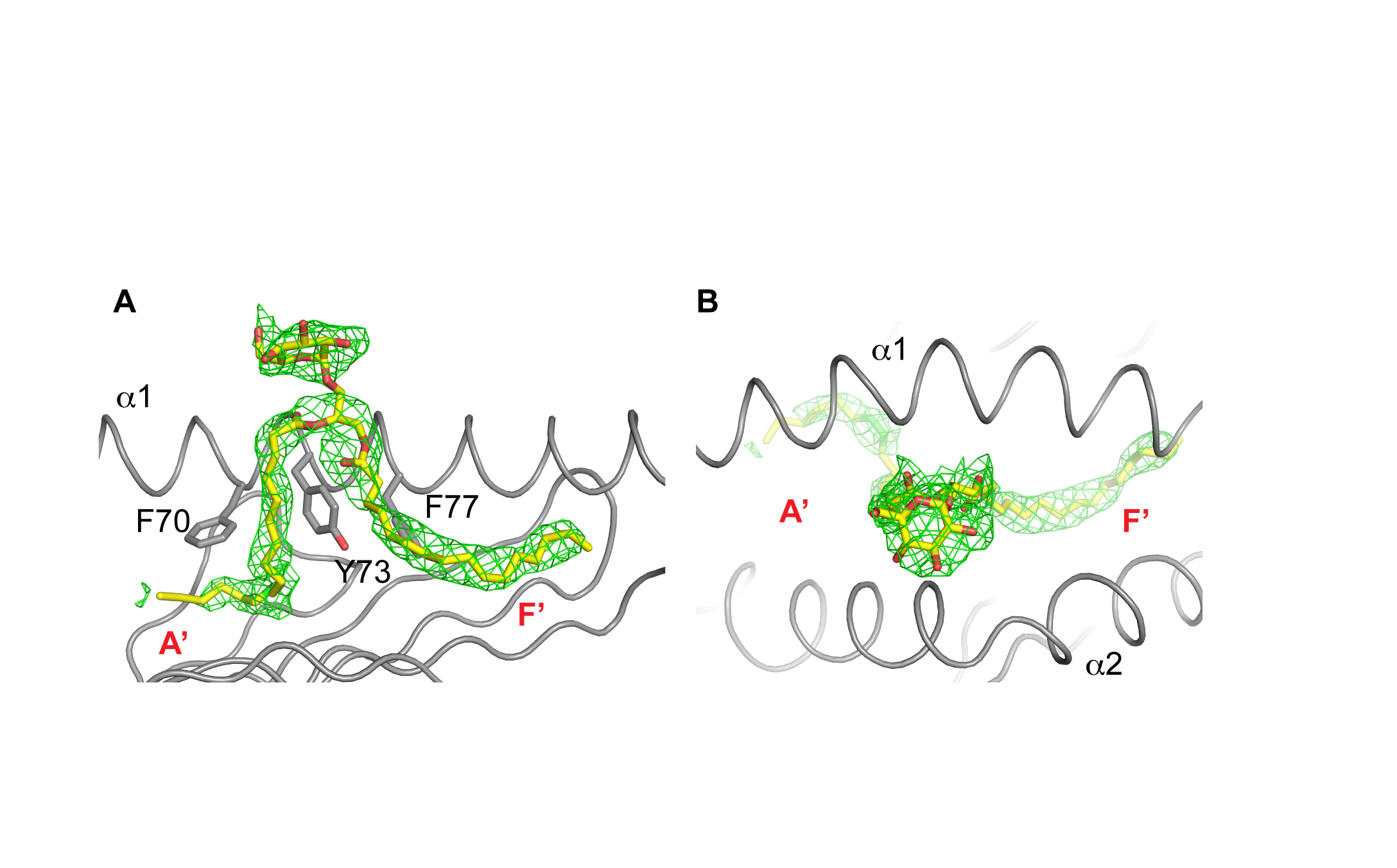

Supplement: Figure S1 — Shake omit map of the Glc-DAG-s2 ligand. Side (A) and top (B) view of the mCD1d binding groove with the ligand in yellow. A shake-omit Fo-Fc map contoured at 2σ is shown as a green mesh around the ligand. (TIF) [file pbio.1001189.s001.tif]

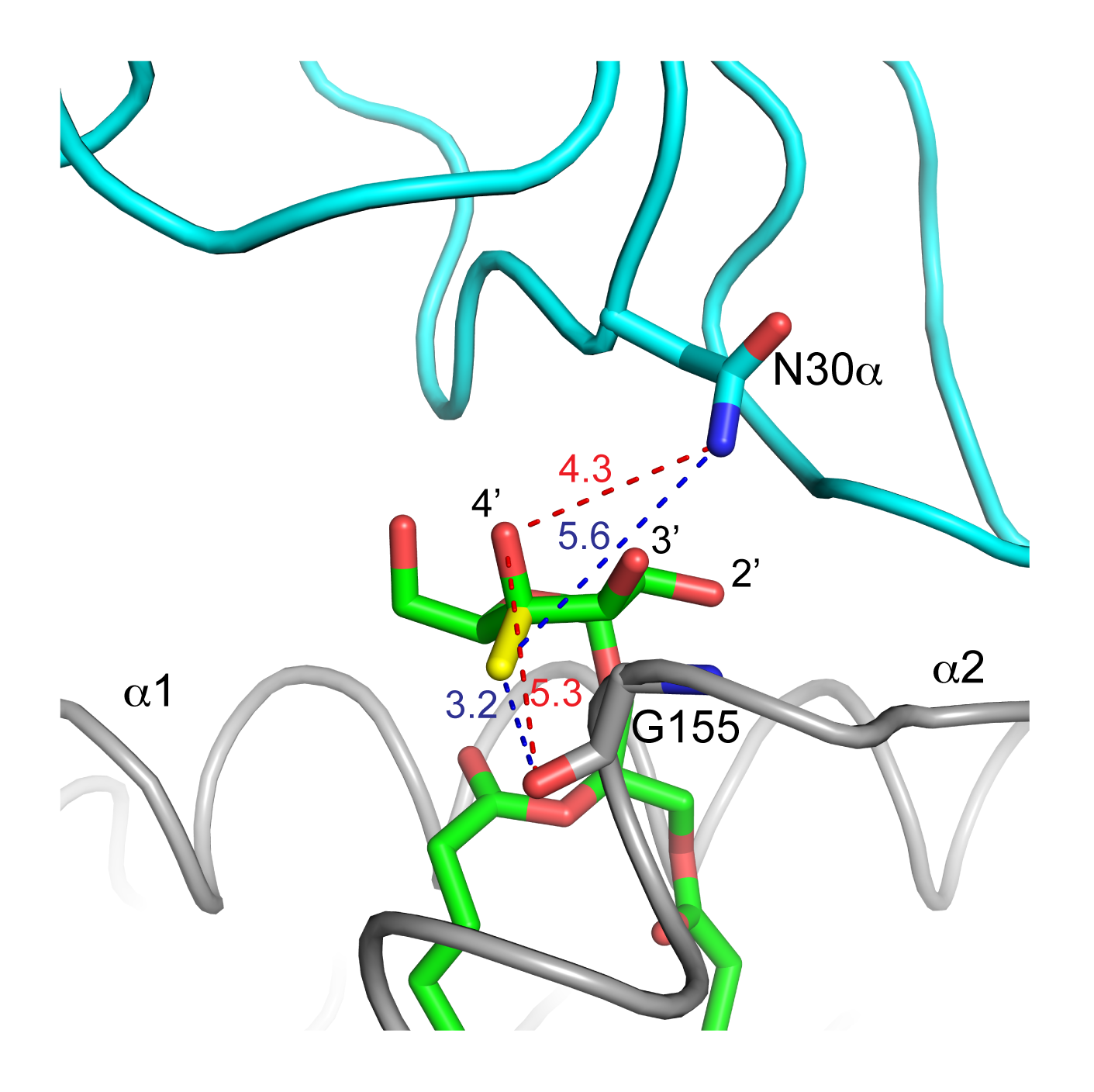

Supplement: Figure S2 — Modeling of Gal-DAG-s2 in the ternary complex. Detailed view of the Gal-DAG-s2 ligand at the mCD1d-TCR interface with the ligand in green, mCD1d in grey, and the iNKT TCR α chain in cyan. The different position of the 4′-OH group for Glc-DAG-s2 is shown in yellow for comparison. Distances between the 4′-OH group of the antigen and Asn30α on the TCR and Gly155 on mCD1d are shown as dashed lines with the corresponding length expressed in Å. (TIF) [file pbio.1001189.s002.tif]
