# Supplementary material for: Unique Interplay between Sugar and Lipid in Determining the Antigenic Potency of Bacterial Antigens for NKT Cells
Source: PLoS Biol. 2011 Nov 1;9(11):e1001189. doi: 10.1371/journal.pbio.1001189 (PMC3206013; doi:10.1371/journal.pbio.1001189)
Supplement: Table S1 — Molecular contacts in the TCR complex. The program CONTACT [26] was used to analyze the molecular interactions within the complexes. Cutoffs of 4 Å (van der Waals interactions), 3.5 Å (hydrogen bonds), and 4.5 Å (salt bridges) were applied. (DOC) [file pbio.1001189.s003.doc]

**Table S1. Molecular contacts in the TCR complex**

| **mCD1d-Glc-DAG-s2-iNKT TCR complex** | | | | | |
| --- | --- | --- | --- | --- | --- |
| **CDR** | **TCR** | **mCD1d** | **Ligand** | **Bonds^a^** |  |
| CDR1α | Thr27 | Val72 |  | VDW |  |
|  | Pro28 | Val72, Ser76 |  | VDW |  |
| CDR3α | Asp94 | Arg79 |  | VDW |  |
|  | Asp94 OD1 | Arg79 NH1,  Arg79 NH2 |  | salt bridge |  |
|  | Asp94 OD2 | Arg79 NH1,  Arg79 NH2 |  | salt bridge |  |
|  | Arg95 | Arg79, Asp80, Ser76 |  | VDW |  |
|  | Arg95 NE | Asp80 OD1,  Asp80 OD2 |  | salt bridge |  |
|  | Arg95 NH1 | Ser76 O  Arg79 NH1,  Arg79 NE |  | H bond |  |
|  | Arg95 NH1 | Asp80 OD1,  Asp80 OD2 |  | salt bridge |  |
|  | Arg95 NH2 | Arg79 NH1 |  | H bond |  |
|  | Gly96 | Asp153, Ala 152 |  | VDW |  |
|  | Gly96 N | Asp153 OD2 |  | H bond |  |
|  | Gly96 O | Ala152 O |  | H bond |  |
|  | Ser97 | Val149 |  | VDW |  |
|  | Leu99 | Val149, Asp80, Glu83, Leu84, Arg79, |  | VDW |  |
|  | Leu99 O | Arg79 NH1 |  | H bond |  |
|  | Gly100 | Arg79 |  | VDW |  |
|  | Arg103 | Arg79 |  | VDW |  |
|  | Arg103 NH1 | Glu83 OE1, Glu83 OE2 |  | salt bridge |  |
| CDR1β | Asn30 | Leu145 |  | VDW |  |
|  | Asn31 | Met87 |  | VDW |  |
| CDR2β | Tyr48 | Glu83, Lys86 |  | VDW |  |
|  | Tyr48 OH | Glu83 OE2, Lys86 NZ |  | H bond |  |
|  | Tyr50 | Glu83, Lys86, Met87 |  | VDW |  |
|  | Tyr50 OH | Glu83 OE2 |  | H bond |  |
|  | Glu56 | Lys86 |  | VDW |  |
|  | Glu56 OE1 | Arg21 NH1, Arg21 NH2 |  | salt bridge |  |
|  | Glu56 OE2 | Arg21 NH1 |  | salt bridge |  |
| CDR3β | Glu96 | Lys148, Ala152 |  | VDW |  |
|  | Glu96 OE1 | Lys148 NZ |  | salt bridge |  |
|  | Glu96 OE2 | Lys148 NZ |  | salt bridge |  |
| CDR1α | Pro28 |  | 5’-O, 6’-O | VDW |  |
|  | Asn30 |  | 3’-O, C-3 | VDW |  |
|  | Asn30 ND2 |  | 3’-O | H bond |  |
| CDR3α | Arg95 |  | 2’-O, glycerol | VDW |  |
|  | Gly96 N |  | 2’-O | H bond |  |
